# Importance of psychological follow-up in rhinoplasty

**DOI:** 10.1016/j.bjorl.2024.101498

**Published:** 2024-09-02

**Authors:** Thales Victor Fernandes Ferreira, Ana Luíza Cézar Fernandes, Mário Pinheiro Espósito

**Affiliations:** a Hospital Otorrino, Cuiabá, MT, Brazil; b Centro Universitário de São Pessoa (UNIPE), João Pessoa, PB, Brazil

**Keywords:** Psychology, Service, Rhinoplasty

## Abstract

**Objective:**

To comprehensively analyze the benefits and harms associated with the use of isotretinoin as a skin preparation agent for rhinoplasty.

**Method:**

A systematic review of the literature was carried out, conducted through a comprehensive search in scientific databases: LILACS and PUBMED.

**Results:**

Although isotretinoin has beneficial effects on skin preparation, some studies indicate that its continuous use before rhinoplasty may interfere with healing, suggesting the need for a period of suspension prior to the procedure. Other concerns include the impact on blood levels, increasing the risk of hematological changes, highlighting the importance of regular hematological monitoring during treatment. The decision to use isotretinoin as preparation for rhinoplasty must take into account the surgeon's experience and the specific characteristics of the patient, seeking to balance the aesthetic benefits with the potential risks.

**Conclusion:**

The decision to use isotretinoin as part of skin preparation for rhinoplasty must be individualized, taking into account the specific characteristics of the patient, their aesthetic objectives and the assessment of risks and benefits. A collaborative approach between the plastic surgeon, dermatologist and patient is essential to ensure safe and effective use of isotretinoin, maximizing its benefits while minimizing the potential harm associated with this therapy.

## Highlights

• Preoperative Emotional Support Manages anxiety and sets realistic expectations.
• Boosts Self-Image and Esteem Improves mental health and patient satisfaction.
• Enhances Postoperative Recovery Helps adapt to changes and cope with recovery.

## Introduction

Rhinoplasty, a surgical procedure aimed at the aesthetic and functional correction of the nose, has gained a prominent place in the search for the desired appearance. However, in addition to the physical aspects, the importance of psychological support throughout the process emerges as a crucial factor for the patient's overall well-being. When considering significant visual transformations, it is imperative to understand the psychological influence that rhinoplasty can have on self-esteem and the perception of one's own image.^1^

The intersection between mental health and cosmetic surgeries, such as rhinoplasty, proves to be a vital area of study. Psychological support, in this context, acts as fundamental support to deal with expectations, anxieties and changes in self-image that may arise before, during and after the procedure. The complexity of emotions associated with physical transformation instigates the need for a holistic approach that embraces both the aesthetic and psychological aspects.^2^

The connection between body image and emotional state is a field of investigation that has expanded considerably. In rhinoplasty, psychological support offers a valuable opportunity to explore and understand the deep motivations behind the search for aesthetic change. Furthermore, it provides a safe space to discuss concerns and realistic expectations, contributing to a more positive and satisfying experience for the patient.^2^

The role of the psychologist in rhinoplasty goes beyond just supporting the patient emotionally. It also involves carefully assessing the individual's psychological readiness for the procedure, identifying possible emotional challenges that may arise during recovery. Understanding the psychological factors involved in decision-making for rhinoplasty allows for a more comprehensive approach, addressing not only aesthetic concerns, but also the psychosocial impacts associated with the intervention.^3^

Contemporary society, which often places a strong emphasis on appearance, requires a critical analysis of the role of rhinoplasty in the construction of personal identity. Psychological support emerges as a crucial tool for deconstructing unrealistic aesthetic standards and promoting a deeper understanding of authenticity and self-acceptance. In this context, psychology plays an essential role in promoting the patient's mental and emotional health, complementing the physical benefits of surgery.^3^

Effective communication between a plastic surgeon and psychologist is vital to ensuring comprehensive patient care. Collaboration between these professionals enables a holistic assessment of the individual's needs, resulting in a more effective and personalized intervention. In this way, the importance of psychological support in rhinoplasty transcends the individual scope, contributing to a more ethical and responsible approach in the field of plastic surgery.

Therefore, the relevance of psychological support in rhinoplasty is undeniable, offering emotional support, critical assessment of motivations and an environment conducive to a deep understanding of the transformations that occur during this process. By integrating the psychological dimension into surgical practice, it is possible not only to improve aesthetic results, but also to promote the patient's mental and emotional well-being, building a more complete and humanized approach to carrying out this procedure.^1^

Therefore, carrying out this study is justified by its scientific importance. This study aims to explore how integrating psychological support before and after rhinoplasty can not only mitigate these emotional challenges, but also promote more positive aesthetic results. By examining the connection between patients' mental health and the perceived success of surgery, this research will contribute to a more holistic approach to surgical practice, highlighting the need to consider not only the physical transformation, but also the promotion of patients' psychological well-being. individuals seeking rhinoplasty.

To comprehensively investigate the importance of psychological support in rhinoplasty, with the aim of understanding the psychosocial and emotional impacts and the influence on patients' post-surgical satisfaction.

## Methods

A systematic literature review was conducted with the purpose of collecting, analyzing and synthesizing all available evidence related to the research topic. A systematic review provides the ability to summarize all existing evidence on a specific topic, covering individual studies, clinical trials, observational research and other types of investigation. This approach contributes to obtaining a comprehensive view of the existing knowledge on the subject.

In order to facilitate data collection, the following guiding question was established: How important is psychological support in rhinoplasty, in order to understand the psychosocial and emotional impacts and the influence on patients' post-surgical satisfaction?

To search for eligible responses, a data collection was carried out through data searches in the scientific databases LILACS and PUBMED, using the descriptors: Psychology, Care and Rhinoplasty, intermediated by the Boolean operator AND.

**Figure 1:**
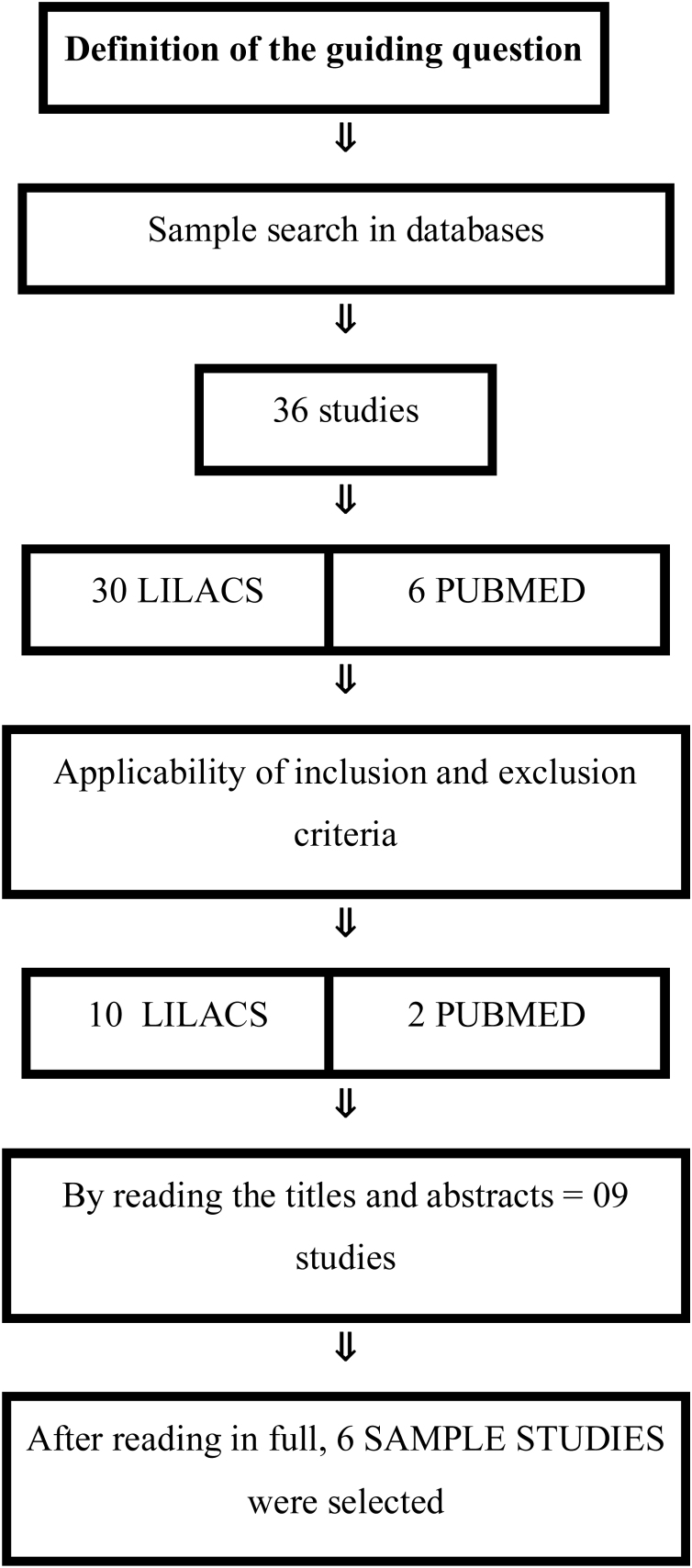
Article selection flowchart. Source: Authors, 2024.

To obtain relevant results, the studies included in the sample were selected according to specific eligibility criteria. Cross-sectional, observational, quantitative, qualitative, cohort research, case reports, experience reports and randomized clinical trials were considered, as long as they were available in full. No specific time frame was defined for the data. Furthermore, studies needed to be accessible in the aforementioned databases and relevant to the research problem in question. On the other hand, review studies, monographs, theses, dissertations and those duplicated in more than one database were excluded. The description of the sample selection was described in Fig. 1.

## Results

The selected sample was organized in Table 1, being structured according to the respective information: Title of the study, author, year of publication, research objective, database and country of origin.

**Table 1:** Title of the study, author, year of publication, research objective, database and country of origin.

Nº	Title	Objective	Author/Year	Data base	Country of origin
1	The impact of preoperative psychological characteristics on postoperative satisfaction and quality of life in patients undergoing septoplasty and inferior turbinate ablation surgery.	To investigate the effect of preoperative mental state on postoperative satisfaction and quality of life in patients undergoing septoplasty and inferior turbinate ablation surgery.	Guven; Gorgulu, 2022	LILACS	Brasil
2	Body dysmorphic and narcissistic personality disorder in cosmetic rhinoplasty candidates.	The aim of this study was to investigate the frequency of BDD and narcissistic personality disorder symptoms in rhinoplasty candidates.	Sahraian et al., 2022	LILACS	Brasil
3	Factors related to postoperative satisfaction in rhinoplasty.	Analyze the association between postoperative quality of life scores and general and psychological characteristics, functional and aesthetic variables to identify the most important determinants of post-rhinoplasty satisfaction.	Souza, 2020	PUBMED	Brasil
4	Assessment of patient's postoperative satisfaction after coverage of the nasal dorsum with the upper lateral cartilage: upper lateral closure.	To evaluate patient satisfaction with the results of vault reconstruction with a technique that uses the upper lateral cartilage to cover the nasal dorsum.	Çağıcı, 2019	LILACS	Brasil
5	Assessment of aesthetic and functional results in rhinoplasty surgery: a prospective study.	Determine patient satisfaction regarding the appearance and function of the nose before and after rhinoplasty surgery with psychological monitoring.	Esteves et al., 2017	LILACS	Brasil
6	Patient assessment of psychosocial dysfunction after nasal reconstruction.	Assess the level of psychosocial suffering associated with nasal reconstruction.	Pimenta et al., 2012	LILACS	Brasil

## Discussion

Rhinoplasty, an aesthetic surgical intervention sought after by those who wish to modify the nasal structure, goes beyond physical changes, and is essential to explore the associated psychosocial and emotional aspects. In analysis of the study by, conducted to understand the impacts on these aspects and their influence on post-surgical satisfaction, highlights the importance of psychological support in this context.^4^

The impact on self-esteem is notable, as the results indicate that psychological support plays a crucial role in managing patients' self-esteem. Pre-surgical psychological interventions contribute to the construction of realistic expectations, minimizing potential postoperative dissatisfaction, in line with previous studies.^5^

When analyzing the influence on expectations, in analysis of the research carried out by it is observed that psychological support has a significant impact on patients' expectations. Effective communication between surgeon and patient, mediated by psychological support, contributes to a clear understanding of the expected results, positively influencing post-surgical satisfaction.^6^

A relevant point discovered in the study by is the reduction of pre-operative anxiety in patients who received psychological support, reinforcing the association between psychological interventions and the reduction of pre-surgical stress, highlighting the importance of the emotional aspect in the surgical process. Post-rhinoplasty psychosocial adaptation emerges as a crucial area, with psychological support helping to manage image changes and the process of adapting to the new facial identity. This investigation reinforces previous conclusions, highlighting the continued need for psychological interventions in the postoperative period.^6^

As mentioned in the data obtained by the general satisfaction of patients after rhinoplasty was evaluated as substantially higher in those who underwent psychological follow-up. Similar studies have indicated that the multidisciplinary approach, including psychological support, contributes to lasting satisfaction, highlighting the importance of emotional well-being in evaluating surgical success. The development of psychological resilience was highlighted as a positive outcome in patients undergoing psychological interventions, playing a crucial role in adapting to physical changes and overcoming post-rhinoplasty psychosocial challenges.^4^

When analyzing the psychological risk factors, presented by it is identified that prior psychological monitoring is associated with a significant reduction in the development of post-surgical psychological disorders, reinforcing the importance of psychological screening in identifying patients susceptible to emotional complications. Thus, the results indicate that psychological support plays a crucial role in rhinoplasty, positively influencing psychosocial and emotional aspects and post-surgical satisfaction. These findings have significant implications for healthcare professionals, highlighting the importance of a multidisciplinary approach to optimize the results and quality of life of patients undergoing this aesthetic intervention.

## Conclusion

In view of the above, research on the importance of psychological support in rhinoplasty highlights the complexity of this aesthetic intervention, going beyond physical modifications to encompass the psychosocial and emotional impacts on patients. The results consistently reveal that psychological support plays a crucial role in managing self-esteem, influencing expectations, reducing pre-operative anxiety, post-rhinoplasty psychosocial adaptation, overall satisfaction and developing psychological resilience.

These findings highlight the imperative need for a multidisciplinary approach, in which healthcare professionals, plastic surgeons and psychologists collaborate to optimize not only physical outcomes, but also patients' quality of life and emotional well-being. However, it is important to recognize the limitations inherent to this research. The study may be influenced by biases, including sample heterogeneity and possible subjectivity in participant responses.

Furthermore, external factors not addressed in the research may impact the results, such as social support outside the clinical context. It is therefore suggested that future research seek to incorporate a more comprehensive approach, considering additional variables that may influence the experience of patients undergoing rhinoplasty.

For future research, it would be valuable to explore the effectiveness of specific psychological interventions at different points in the surgical process, from the consultation phase to the late postoperative period. Furthermore, longitudinal investigations could provide deeper insights into the evolution of patients' satisfaction and emotional well-being over time. A deeper understanding of these aspects would allow the development of personalized psychological support protocols, adapted to the individual needs of patients, contributing to a more holistic and effective approach to rhinoplasty and similar procedures.

## Funding

The article in question has no funding.

## Conflicts of interest

The authors declare no conflicts of interest.
